# The Story of the Insane from Year to Year

**Published:** 1904-02-20

**Authors:** 


### THE STORY OF THE INSANE FROM YEAR TO YEAR.

Sixteenth Annual Series.

### ROYAL LUNATIC HOSPITAL, MANCHESTER.

On the first of January the hospital contained 318 certified patients and 31 voluntary boarders. At the end of the year the total number in the house was 360. Of the certified cases 48 were discharged recovered, 23 were discharged relieved, and 26 died. Of the boarders 35 were discharged during the year, and 6 were certified as patients. The total number under treatment during the year was 498. The percentage of recoveries calculated on the admitted certified patients was 45·7, and the percentage of deaths calculated on the total number under treatment was 6·1. Of the year's deaths 19 were caused by cerebral and spinal diseases, 1 by pneumonia, and 2 by phthisis. Of the year's admissions 36 owed the insanity to domestic trouble and mental worry, 14 to hereditary influence, and 16 to intemperance in drink. The Chairman, in his report, draws attention to the "importance of the institution for the reception of educated patients who are unable to provide from their own property the full normal charges, and for whom the only alternative would be residence in the county asylum. It is difficult to estimate how much more distressing to themselves and friends these cases would be if so transferred, and the diminution in the chances of recovery in curable cases." This is a point which The Hospital has never lost sight of in its notices of asylums, but we miss from the statistics of the Royal Lunatic Hospital a table showing the number of patients received at low rates and the number received at high rates of payment, so that we have no means of judging to what extent it is really a charity, or to what extent it is competing with proprietary private asylums to secure rich patients. It is to be hoped that these details will be supplied in the next annual report. When referring to the deaths from consumption, Dr. Mould says that "modern research and treatment have shown the necessity of strict isolation in these cases, and it is a matter for consideration how they may be treated in an entirely separate building." Doubtless annexes for phthisical patients will soon be deemed as essential at our large asylums as infectious hospitals are at present. Both the stewards and the matron retired during the year, and both received pensions; but the amount granted is not stated.

### EAST RIDING ASYLUM, BEVERLEY.

On the first of January this asylum contained 449 patients, and at the end of the year the number had risen to 457. There were 84 first admissions, 17 readmissions, 30 discharged recovered, 10 discharged not improved, and 50 died. The total number under treatment was 550, and the average daily number resident was 455. The recoveries were

38·46 per cent. of the admissions, and the deaths were 10·97 per cent. of the average number resident. Of the deaths during the year 12 were caused by cerebral and spinal diseases, 3 by pneumonia, 1 by colitis, and 8 by consumption. Of the year's admissions the insanity was supposed to be caused by moral causes in 9 instances, and among the physical causes drink accounts for 9 and hereditary influence for 29. The asylum is being added to by a block for 120 male patients and provision for 25 private patients is also being made. The contract was £22,845, but this seems to include a new dining-hall and other extensions. Enteric fever broke out, and 3 female patients were attacked. The cause of the outbreak was not ascertained. The weekly cost of maintenance was 7s. 10½d. per head.

### WARNEFORD ASYLUM, OXFORD.

THIS lunatic hospital contained 92 patients on the first of January and 93 on the last day of the year. There were 26 first admissions and 5 readmissions. The recoveries were 15 and the deaths 7. The total number under treatment was 123, and the average number resident was 95. The recoveries reached the very creditable percentage of 53·6 of the admissions, and the deaths were 7 4 on the average number resident. Five of the year's admissions had their insanity caused by moral causes, 2 by drink, and 9 by hereditary influence. This asylum is one of its class which has tried to do its duty to those patients who were unable to pay high maintenance rates and for whom such institutions were originally provided, and we therefore much regret to learn that the revenue from the Warneford bequests has diminished so much that it has been found necessary to increase some of the payments made by patients, and although only 20 were interfered with and none of these exceeded 5s. a week, we can well understand how painful this step must have been to the committee. It speaks ill for the people of the county and city of Oxford that they have not come forward and provided the necessary money either by donations or by annual subscriptions. We trust this blot will soon be wiped out. The average weekly cost per head is nearly 32s. The committee publish a table showing the amount of charitable relief given by the asylum, and from it we learn that one patient paid only 5s. 9d. a week, another only 6s. 6d., 12 paid only 10s. a week, and 20 paid £1 a week or less. These rates were for a year at least, but many others paid small sums for periods of less than a year. We earnestly commend this institution to those who are able and willing to help in a really charitable work.

### DUNDEE ROYAL ASYLUM.

AT the beginning of the year there were in residence 419 patients, and by the end of the year this number had risen to 471. The average number resident was 436, and the total number under treatment was 614. There were 155 first admissions, 46 readmissions, 59 recoveries, 41 discharged relieved, and 40 deaths. Calculated on the admissions the recoveries work out at 29·35 per cent., and calculated on the average number resident the deaths were 9·16 per cent. Of the year's deaths 22 were caused by cerebral and spinal diseases, 1 by pneumonia, and only 2 by consumption. Among the moral causes of insanity in those admitted we notice that mental anxiety figures 28 times, adverse circumstances 3 times, and religion 5 times. And among the physical causes, drink, heredity and previous attacks are responsible for the majority, being respectively 80, 33 and 63. Dr. Rorie once more emphasises the importance of early treatment, and he says that he is "still much handicapped by the delay arising from legal formalities and other causes preventing the patients from receiving treatment at an early date." He thinks that until lately not much progress has been made in the direction of obtaining this early treatment, although attention has been often turned to it. It is pointed out that as early as the year 1822 the medical report of the Dundee Asylum stated that the number of cures holds a direct ratio to the recency of the attack, and yet this well-known fact has to be reiterated periodically by almost every medical superintendent in the country. The ideal course is foreshadowed somewhat as follows:—(1) The establishment of conveniently situated dispensaries where patients could obtain advice and treatment; (2) special wards in general hospitals to be set apart for acute and transient cases; and (3) the heterogeneous mixture of cases found in asylums at present should be more thoroughly broken up, so that the idiots would have a branch to themselves and the epileptics another, and (what we look upon as most important) those cases who oscillate between the prison and the asylum should be kept rigidly apart from the others. The asylum has been sold to the District Board of Lunacy for the sum of £90,000, and it has been resolved by the committee of the Royal Asylum to buy or to build an asylum with this money for the cure of the insane. We may therefore soon see the erection of a model institution embodying all the improvements which Dr. Rorie's great experience can suggest.

### EAST SUSSEX ASYLUM AT HAYWARD'S HEATH.

ON the first of January this asylum contained 975 patients, and on December 31st the number had risen to 983. There were 217 first admissions, 36 readmissions, 73 discharged recovered, 24 discharged relieved, 49 discharged not improved, and 99 died. The total number under treatment was 1,220, and the average daily number resident was 993. Calculated on the admissions the recoveries were 30·2 per cent., and calculated on the average number resident the deaths were 9·9 per cent. Of the year's deaths 41 were caused by cerebral and spinal diseases, 8 by pneumonia and bronchitis, 7 by colitis, and 10 by consumption. Of the year's admissions 29 were stated to owe their insanity to various moral causes; and among the physical causes, hereditary influence, previous attacks, and intemperance in drink were the most potent, accounting respectively for 61, 62, and 21. The value of this table is, however, considerably reduced as 72 are put down as of unknown origin. At the date of the report there was considerable pressure on the space, and in March, 1902, the asylum contained upwards of 1,000 patients. Since then the opening of the new asylum at Hellingly will have relieved this overcrowding, and henceforth Hayward's Heath will be given over entirely to the patients from the Borough of Brighton. Small-pox broke out during the year, the patient being a nurse who had joined the staff 12 days previously. She was promptly isolated and the outbreak was confined to this single case. Mr. Lenton, the storekeeper, and two attendants were pensioned during the year, but the amounts received by these officials is not stated. The committee say they "have again the pleasure to record their appreciation of the zeal and ability with which Dr. Walker discharges the duties of medical superintendent." The weekly cost of maintenance per head is 9s. 8¼d.

### LONDON COUNTY ASYLUM AT CANE HILL.

AT the beginning of the year there were 2,170 patients in residence, and at the end of 1902 the number had fallen to 2,126. There were 277 first admissions, 46 readmissions, 132 recoveries, 53 discharged relieved, 24 discharged not improved, and 156 died. The total number under treatment was 2,493, and the average daily number resident was 2,136. The recoveries were 42·17 per cent. of the admissions, and the deaths were 7 38 per cent. of the average number resident; the first of these percentages is above the average and the second is below it. Of the year's deaths 72 were caused by cerebral and spinal diseases, 15 by pneumonia and bronchitis, 23 by consumption and 5 by dysentery. Of the year's admissions such moral causes as domestic trouble, adverse circumstances and religion account for 132, and among the physical causes we note drink as producing the insanity in 80 instances, hereditary influence in 57, and previous attacks in 98. Lectures continue to be given to the staff on first aid and nursing. During the year 23 of the attendants and nurses obtained the St. John's Ambulance certificate, and 5 obtained the certificate of the Medico-Psychological Association. The committee say that the high standard of efficiency at this asylum, and the smoothness of its administration reflect the highest credit on Dr. Moody and the other members of the asylum staff. The weekly rate of maintenance is nearly 11s. 9d. a head.

### LANARK DISTRICT ASYLUM AT HARTWOOD.

THERE were 125 first admissions, 78 readmissions, and 18 unknown whether they had previously been under treatment or not. The recoveries were 83, the discharged relieved 22, the deaths 52, and at the end of the year 870 remained in the asylum. The total number under treatment was 1,028, and the average daily number resident was 834. The recoveries stand at 37·5 per cent. of the admissions, and the deaths at 6 2 per cent. of the average number resident. The former percentage is about the average of kindred institutions, and the latter is considerably below it. The asylum is nearly full, but there are 150 cases chargeable to Edinburgh and to Renfrewshire and also 34 private patients, the latter, we are glad to hear, being received at low rates of board. Of the year's deaths 14 were caused by cerebral and spinal diseases, 6 by consumption, 3 by pneumonia, and 3 by enteritis. Of the year's admissions, 22 were supposed to owe their insanity to various moral causes, and hereditary influence alone or combined with other causes accounts for 41. Intemperance in drink is responsible for 37. Dr. Kerr thinks, and undoubtedly he is right, that if an accurate history of the admissions could be obtained, heredity would be found to be a much more potent cause than the above numbers would lead one to suppose. The attendants and nurses are systematically trained for their duties, but unfortunately the course is only of two years' duration. Twelve attendants and nurses obtained the certificate of the Medico-Psychological Association, and a bonus of £2 is given by the committee to everyone who holds this certificate. This is a wise provision. The committee "record their appreciation of the successful results of Dr. Kerr's painstaking and able services in the superintendence of the Asylum." The weekly rate of maintenance is 9s. 4d. per head.

### COLCHESTER ASYLUM FOR IDIOTS.

ON the first of January this institution contained 252 patients; 36 were admitted during the year, 12 were discharged, and 24 died. The average daily number resident was 250, being 4 higher than the previous year. Mr. Turner was able to report that the newly-admitted patients were more favourably placed, as regards chances of improvement, than those usually sent in. In fact 23 of them were sent to the schools, workshops, and garden, and they will there acquire knowledge which will be useful to them when discharged. As is usual in idiot asylums the death-rate from consumption is very high, and assuredly some means should be devised of separating those suffering from tubercular diseases from the healthy. Apart from this drawback the asylum is very well equipped, and it does an immense amount of good. The governors wisely decided to buy an additional 6 acres of land adjoining that at present owned by them, and when it is realised that the asylum is in the town of Colchester, the value of this land to the institution can hardly be over-estimated. The purchase has, however, entirely exhausted the reserve fund.

